# Supplementary material for: Targeted Control of Gene Expression Using CRISPR-Associated Endoribonucleases
Source: Cells. 2025 Apr 3;14(7):543. doi: 10.3390/cells14070543 (PMC11988398; doi:10.3390/cells14070543)
Supplement: Supplementary file 1 [file cells-14-00543-s001.zip › cells-3505255-supplementary.pdf]

Supplemental Materials
for

Targeted Control of Gene Expression Using CRISPR-Associated Endoribonucleases

Sagar J. Parikh [1], Heather M. Terron [1], Luke A. Burgard [1,2], Derek S. Maranan [1,2], Dylan D. Butler [1,2], Abigail Wiseman [1], Frank M. LaFerla [1,2], Shelley Lane [1] and Malcolm A. Leissring [1,*]

[1] Institute for Memory Impairments and Neurological Disorders, University of California, Irvine, CA 92697, USA
[2] Department of Neurobiology and Behavior, University of California, Irvine, CA 92697, USA
* Correspondence: m.leissring@uci.edu

Contents

pp.	Figure/Table	Title
2	Figure S1	Effects of nuclear localization and nuclear exclusion signals on the performance of 3' DREDGE using dCas12a.
3	Figure S2	Overview of RNA-seq sequencing statistics.
4	Figure S3	Volcano plots for all RNA-seq pairwise comparisons.
5-6	Figure S4	Top results of BLASTN search for Cas12a DR within the murine RefSeq mRNA database.
7	Figure S5	Overview of genotyping results confirming the successful introduction of three Cas12a DRs into the 3' UTR of murine *CTSD* via CRISPR-Cas.
8	Figure S6	Overview of genotyping results confirming the successful introduction of the DR+TG insert into the 3' region of murine *CTSD* via CRISPR-Cas.
9-10	Supp. Methods	Cloning methods for individual DNA constructs.
10	Supp. References	References for *Supplemental Methods*.

Supplementary Figure S1.

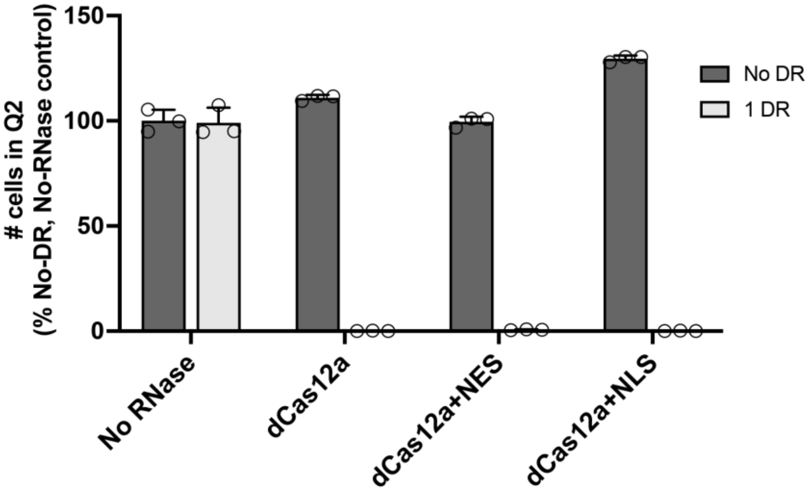

Figure S1. Effects of nuclear localization and nuclear exclusion signals on the performance of 3' DREDGE using dCas12a. Graph of the percentage of cells in Q2 (representing mCherry+ cells that are also GFP+) in MEFs transiently co-transfected with pCAG-GFPd2 expression vectors containing either 0 (No DR) or 1 DR, together with vectors expressing mCherry alone (No RNase) or together with dCas12a with no nuclear localization or exclusion signals (dCas12a), dCas12a with a nuclear exclusion sequence (dCas12a+NES), or dCas12a with 2 nuclear localization sequences (dCas12a+NLS). All data were normalized to cells transiently co-transfected with No-DR GFPd2 and No-RNase mCherry vectors. Data are mean ± SEM for 3 replications per condition.

Supplementary Figure S2. Overview of RNA-seq sequencing statistics.

Library Sample ID Conversion Key

1DR-hdC:	GFPd2 w/ 1 DR + dCas12a
1DR-MT:	GFPd2 w/ 1 DR + No RNase
V-hdC:	GFPd2 w/ No DR + dCas12a
V-MT:	GFPd2 w/ No DR + No RNase

AZENTA RNA-Seq Analysis Report

Project	Sample ID	Barcode Sequence	# Reads	Yield (Mbases)	Mean Quality Score	% Bases >= 30
30-1159494819	1DR-hdC-1	CACGCAAT +ATGGAAGG	41,531,722	12,460	38.81	94.24
30-1159494819	1DR-hdC-2	GGAATGTC +GTGTTCCT	33,539,137	10,062	38.40	92.41
30-1159494819	1DR-hdC-3	TGGTGAAG +GCTGTAAG	51,331,218	15,399	38.41	92.54
30-1159494819	1DR-MT-1	TAGGAGCT +GTTAAGGC	21,052,897	6,316	38.67	93.56
30-1159494819	1DR-MT-2	TACTCCAG +CCTATACC	34,995,252	10,499	38.45	92.69
30-1159494819	1DR-MT-3	CGGCATTA +TGACTGAC	56,506,993	16,952	38.76	94.03
30-1159494819	V-hdC-1	TACGGTCT +GCAATGGA	25,283,748	7,585	38.76	93.99
30-1159494819	V-hdC-2	AAGACCGT +CAATCGAC	32,089,836	9,627	38.80	94.13
30-1159494819	V-hdC-3	CAGGTTCA +GGCGTTAT	29,279,987	8,784	38.53	92.93
30-1159494819	V-MT-1	TTACCGAC +CGTATTCG	35,550,039	10,665	38.52	92.96
30-1159494819	V-MT-2	TCGTCTGA +TCAAGGAC	10,192,202	3,058	38.14	91.27
30-1159494819	V-MT-3	TTCCAGGT +AAGCACTG	30,474,621	9,142	38.51	92.98

Project	# Reads	Yield (Mbases)	Mean Quality Score	% Bases >= 30
30-1159494819	401,827,652	120,549	38.59	93.27

Sample ID	Total Reads	Reads mapped to reference	Percent reads mapped to reference
1DR-hdC-1	82,627,326	61,302,484	74.19
1DR-hdC-2	66,772,616	51,037,701	76.44
1DR-hdC-3	102,294,784	78,608,990	76.85
1DR-MT-1	41,733,612	31,736,287	76.04
1DR-MT-2	69,708,990	53,871,280	77.28
1DR-MT-3	112,554,558	86,668,701	77.00
V-hdC-1	50,301,080	38,441,813	76.42
V-hdC-2	63,733,124	48,244,890	75.70
V-hdC-3	58,114,118	43,570,273	74.97
V-MT-1	70,745,040	53,606,786	75.77
V-MT-2	20,102,072	15,689,720	78.05
V-MT-3	60,640,238	46,829,118	77.22

Note: All data from the RNA-seq analyses can be accessed online at: https://osf.io/kumjr.

Supplementary Figure S3. Volcano plots for all RNA-seq pairwise comparisons.

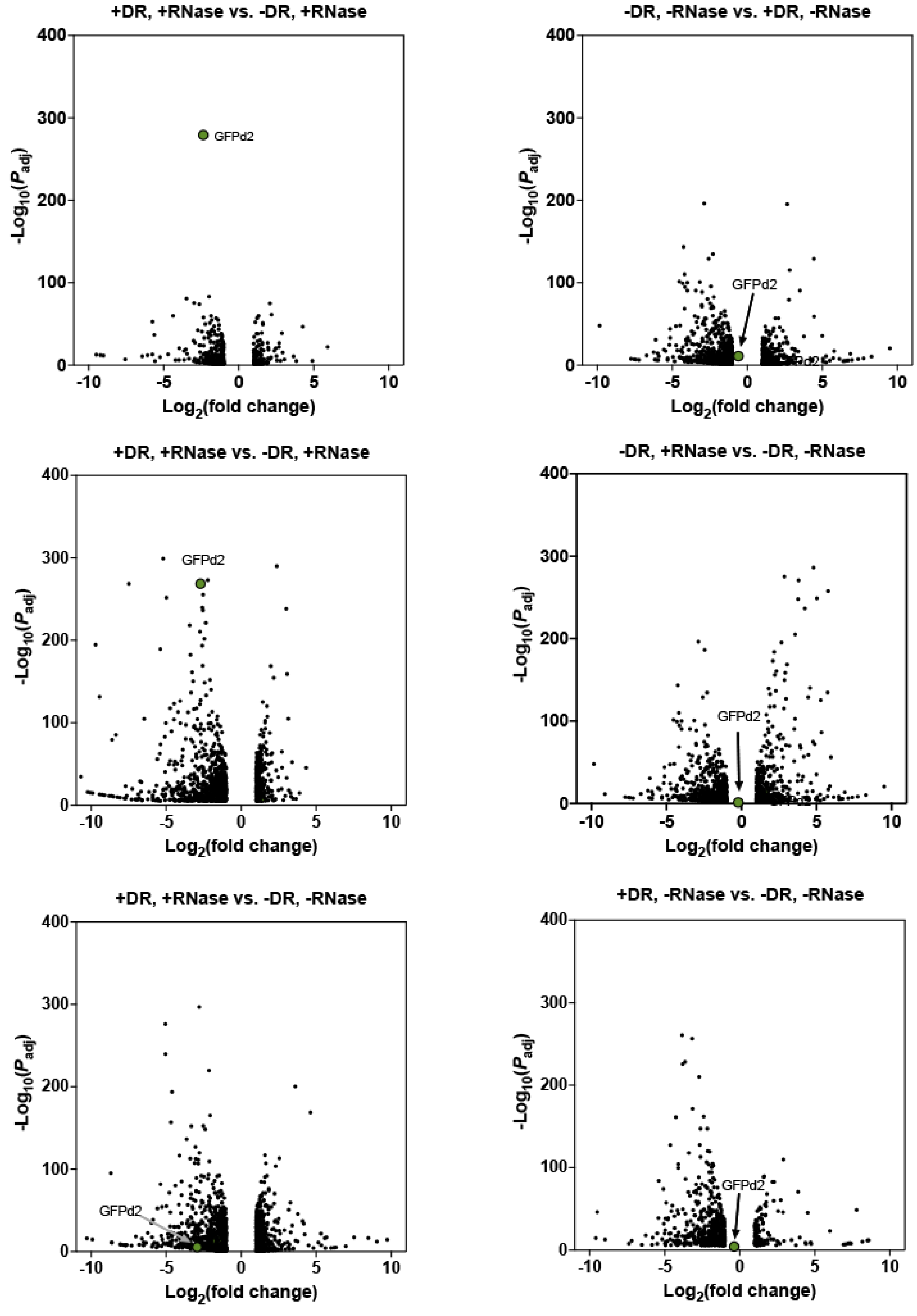

Note: All data from the RNA-seq analyses can be accessed online at: https://osf.io/kumjr.

Supplementary Figure S4. Top results of BLASTN search for Cas12a DR within the murine RefSeq mRNA database.

A

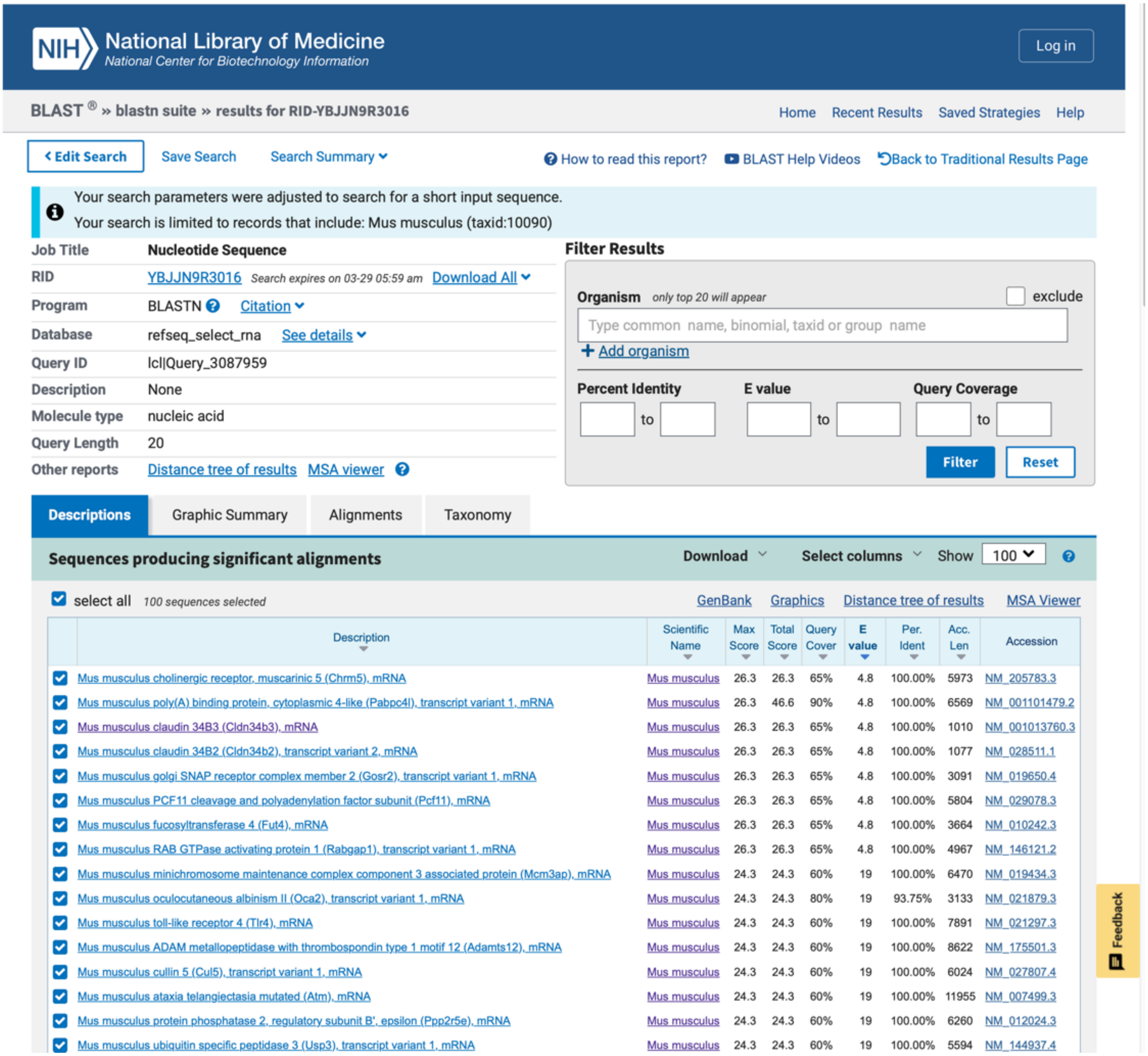

Description	Scientific Name	Max Score	Total Score	Query Cover	E value	Per. Ident	Acc. Len	Accession
Mus musculus cholinergic receptor, muscarinic 5 (Chrm5), mRNA	Mus musculus	26.3	26.3	65%	4.8	100.00%	5973	NM_205783.3
Mus musculus poly(A) binding protein, cytoplasmic 4-like (Pabpc4l), transcript variant 1, mRNA	Mus musculus	26.3	46.6	90%	4.8	100.00%	6569	NM_001101479.2
Mus musculus claudin 34B3 (Cldn34b3), mRNA	Mus musculus	26.3	26.3	65%	4.8	100.00%	1010	NM_001013760.3
Mus musculus claudin 34B2 (Cldn34b2), transcript variant 2, mRNA	Mus musculus	26.3	26.3	65%	4.8	100.00%	1077	NM_028511.1
Mus musculus golgi SNAP receptor complex member 2 (Gosr2), transcript variant 1, mRNA	Mus musculus	26.3	26.3	65%	4.8	100.00%	3091	NM_019650.4
Mus musculus PCF11 cleavage and polyadenylation factor subunit (Pcf11), mRNA	Mus musculus	26.3	26.3	65%	4.8	100.00%	5804	NM_029078.3
Mus musculus fucosyltransferase 4 (Fut4), mRNA	Mus musculus	26.3	26.3	65%	4.8	100.00%	3664	NM_010242.3
Mus musculus RAB GTPase activating protein 1 (Rabgap1), transcript variant 1, mRNA	Mus musculus	26.3	26.3	65%	4.8	100.00%	4967	NM_146121.2
Mus musculus minichromosome maintenance complex component 3 associated protein (Mcm3ap), mRNA	Mus musculus	24.3	24.3	60%	19	100.00%	6470	NM_019434.3
Mus musculus oculocutaneous albinism II (Oca2), transcript variant 1, mRNA	Mus musculus	24.3	24.3	80%	19	93.75%	3133	NM_021879.3
Mus musculus toll-like receptor 4 (Tlr4), mRNA	Mus musculus	24.3	24.3	60%	19	100.00%	7891	NM_021297.3
Mus musculus ADAM metallopeptidase with thrombospondin type 1 motif 12 (Adamts12), mRNA	Mus musculus	24.3	24.3	60%	19	100.00%	8622	NM_175501.3
Mus musculus cullin 5 (Cul5), transcript variant 1, mRNA	Mus musculus	24.3	24.3	60%	19	100.00%	6024	NM_027807.4
Mus musculus ataxia telangiectasia mutated (Atm), mRNA	Mus musculus	24.3	24.3	60%	19	100.00%	11955	NM_007499.3
Mus musculus protein phosphatase 2, regulatory subunit B', epsilon (Ppp2r5e), mRNA	Mus musculus	24.3	24.3	60%	19	100.00%	6260	NM_012024.3
Mus musculus ubiquitin specific peptidase 3 (Usp3), transcript variant 1, mRNA	Mus musculus	24.3	24.3	60%	19	100.00%	5594	NM_144937.4

Supplementary Figure S4 (Cont'd). Top results of BLASTN search for Cas12a DR within the murine RefSeq mRNA database.

B

Download GenBank Graphics — Next Previous Descriptions

Mus musculus cholinergic receptor, muscarinic 5 (Chrm5), mRNA

Sequence ID: NM_205783.3 Length: 5973 Number of Matches: 1

Range 1: 4858 to 4870 GenBank Graphics — Next Match Previous Match

Score	Expect	Identities	Gaps	Strand
26.3 bits(13)	4.8	13/13(100%)	0/13(0%)	Plus/Minus

```
Query  2     ATTTCTACTAAGT  14
             |||||||||||||
Sbjct  4870  ATTTCTACTAAGT  4858
```

Related Information
Gene - associated gene details
Genome Data Viewer - aligned genomic context

Download GenBank Graphics Sort by: E value — Next Previous Descriptions

Mus musculus poly(A) binding protein, cytoplasmic 4-like (Pabpc4l), transcript variant 1, mRNA

Sequence ID: NM_001101479.2 Length: 6569 Number of Matches: 2

Range 1: 4133 to 4145 GenBank Graphics — Next Match Previous Match

Score	Expect	Identities	Gaps	Strand
26.3 bits(13)	4.8	13/13(100%)	0/13(0%)	Plus/Minus

```
Query  1     AATTTCTACTAAG  13
             |||||||||||||
Sbjct  4145  AATTTCTACTAAG  4133
```

Range 2: 4666 to 4675 GenBank Graphics — Next Match Previous Match First Match

Score	Expect	Identities	Gaps	Strand
20.3 bits(10)	297	10/10(100%)	0/10(0%)	Plus/Plus

```
Query  9     CTAAGTGTAG  18
             ||||||||||
Sbjct  4666  CTAAGTGTAG  4675
```

Related Information
Gene - associated gene details
Genome Data Viewer - aligned genomic context

Download GenBank Graphics — Next Previous Descriptions

Mus musculus claudin 34B3 (Cldn34b3), mRNA

Sequence ID: NM_001013760.3 Length: 1010 Number of Matches: 1

Range 1: 723 to 735 GenBank Graphics — Next Match Previous Match

Score	Expect	Identities	Gaps	Strand
26.3 bits(13)	4.8	13/13(100%)	0/13(0%)	Plus/Plus

```
Query  3    TTTCTACTAAGTG  15
            |||||||||||||
Sbjct  723  TTTCTACTAAGTG  735
```

Related Information
Gene - associated gene details
Genome Data Viewer - aligned genomic context

Download GenBank Graphics — Next Previous Descriptions

Mus musculus claudin 34B2 (Cldn34b2), transcript variant 2, mRNA

Sequence ID: NM_028511.1 Length: 1077 Number of Matches: 1

Range 1: 722 to 734 GenBank Graphics — Next Match Previous Match

Score	Expect	Identities	Gaps	Strand
26.3 bits(13)	4.8	13/13(100%)	0/13(0%)	Plus/Plus

```
Query  3    TTTCTACTAAGTG  15
            |||||||||||||
Sbjct  722  TTTCTACTAAGTG  734
```

Related Information
Gene - associated gene details
Genome Data Viewer - aligned genomic context

Feedback

Supplementary Figure S5.

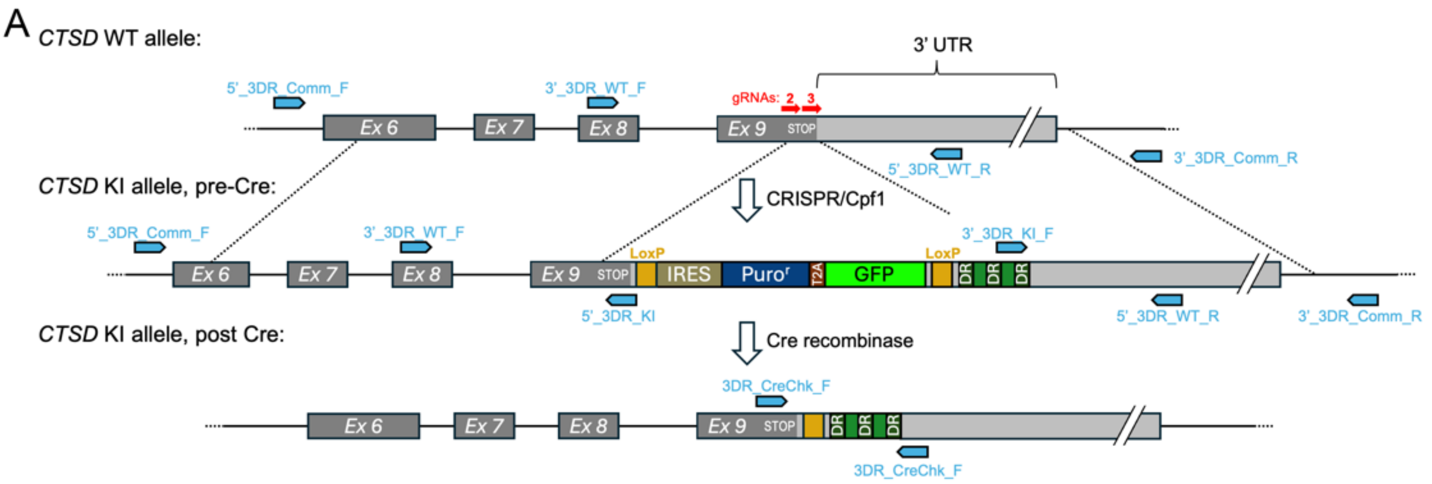

B gRNA design and alterations to knock-in insert:

	gRNA_2	gRNA_3
**gRNA sequence**	**ACAGAGACAACAATAGGGTCG**	**CCAATGCTGTCGTACTCTAAC**
**Endog. sequence**	...TTTGACAGAGACAACAATAGGGTCGGC...	...TTTGCCAATGCTGTCGTACTCTAACTT...
**A.A. sequence**	PheAspArgAspAsnAsnArgValGly	PheAlaAsnAlaValValLeuSTP
**KI insert sequence**	...TTCGATAGGGACAACAACAGAGTGGGC...	...TTCGCGAACGCCGTAGTCCTGTGATAA...

C Genotyping primers:

Primer Name	Primer Sequence
5'_3DR_Comm_F	CTGATCACTTACTTCCTCCTAGG
5'_3DR_WT_R	CTCAGCAGCTATTAAACCAGTAGG
5'_3DR_KI_R	CACTCTGTTGTTGTCCCTATCG
3'_3DR_Comm_R	ATATTCTCCACAGCCAGCGTG
3'_3DR_WT_F	AGACAAGTATATACTCAAGGTGAGTCG
3'_3DR_KI_F	CAAGATTTGCCAAAGCGCTTG
3DR_CreChk_F	CGATAGGGACAACAACAGAGTGG
3DR_CreChk_R	GCTTTGGCAAATCTTGATCTACAC

D Genotyping results:

Amplicon	Forward Primer	Position in WT	Position in KI, pre-Cre	Position in KI, post-Cre	Reverse Primer	Position in WT	Position in KI, pre-Cre	Position in KI, post-Cre	Size of Correct Amplicon (bp)	Result
5' WT	5'_3DR_Comm_F	979	979	-	5'_3DR_WT_R	2161	-	-	1182	no amplicon
5' KI	5'_3DR_Comm_F	979	979	-	5'_3DR_KI_R	4387	1858	-	879	100% match by Sanger sequencing
3' WT	3'_3DR_WT_F	1605	1605	-	3'_3DR_Comm_R	2660	4886	-	1056	no amplicon
3' KI	3'_3DR_KI_F	-	4095	-	3'_3DR_Comm_R	-	4886	-	792	100% match by Sanger sequencing
Cre Check	3DR_CreChk_F	-	1837	1837	3DR_CreChk_R		3863	2072	235	100% match by Sanger sequencing

Figure S5. Overview of genotyping results confirming the successful introduction of three Cas12a DRs into the 3' UTR of murine *CTSD* via CRISPR-Cas. **A**, Genomic structure of the mouse *CTSD* gene prior to modification (top), after introduction of the "gene-trap" knockin (KI) allele (middle), and after removal of all elements besides the 3 Cas12a DRs using Cre-recombinase (bottom). The approximate positions of genotyping primers are indicated (blue arrows). **B**, Table showing the gRNA sequences using for CRISPR-Cas, the targeted endogenous sequence (with PAM sequence in red), the amino-acid sequences encoded by the sequence targeted by the gRNAs, and the modifications to gRNA-targeted regions within the KI targeting construct. **C**, Table showing the sequences for the DNA primers used for genotyping (with positions indicated in blue in panel **A** and identified numerically in panel **D**). **D**, Summary of genotyping results, showing the relative positions of individual primers, the predicted amplicon sizes for primer pairs, and the outcome of PCR amplification and subsequence Sanger sequencing. These results apply to a single clonal cell line chosen for use in downstream experiments. Note that no amplification was obtained for WT amplicons.

Supplementary Figure S6.

A

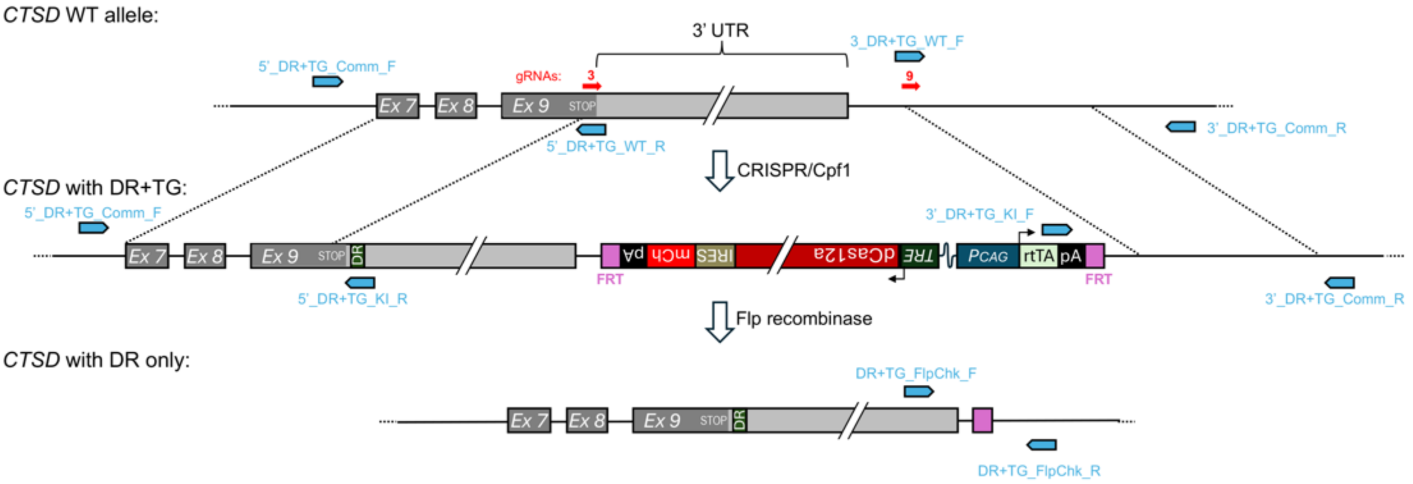

B
gRNA design and alterations to knock-in insert:

	gRNA_3	gRNA_9
**gRNA sequence**	**CCAATGCTGTCGTACTCTAAC**	**TGAATTGGGACCTTGGCGACG**
**Endog. sequence**	...TTTGCCAATGCTGTCGTACTCTAACTT...	...TTTCTGAATTGGGACCTTGGCGACG...
**A.A. sequence**	PheAlaAsnAlaValValLeuSTP	N/A
**KI insert sequence**	...TTCGCGAACGCCGTAGTCCTGTGATAA...	deleted and replaced w/ FRT site

C
Genotyping primers:

Primer Name	Primer Sequence
5'_DR+TG_Comm_F	CTAGACAAGCTATGCCCTATACTCAC
5'_DR+TG_WT_R	GTTAGAGTACGACAGCATTGGC
5'_DR+TG_KI_R	GGAGCAAATCTACACTTAGTAGAAATTATTTTTTACTTATCAC
3'_DR+TG_Comm_R	GATGCTTGTAGAACAACTGTAGTCC
3'_DR+TG_WT_F	TTTCTGAATTGGGACCTTGGCG
3'_DR+TG_KI_F	GATGCTTGTAGAACAACTGTAGTCC
DR+TG_FlpChk_F	CAGACAAGTATATACTCAAGGTGAGTCG
DR+TG_FlpChk_R	AGTTCTAGTTCAGACTCATCTTGCC

D
Genotyping results:

Amplicon	Forward Primer	Position in WT	Position in KI, pre-Flp	Position in KI, post-Flp	Reverse Primer	Position in WT	Position in KI, pre-Flp	Position in KI, post-Flp	Size of Correct Amplicon (bp)	Result
5' WT	5'_DR+TG_Comm_F	1219	1219	-	5'_DR+TG_WT_R	1886	-	-	668	no amplicon
5' KI	5'_DR+TG_Comm_F	1219	1219	-	5'_DR+TG_KI_R	-	1924	-	706	100% match by Sanger sequencing
3' WT	3'_DR+TG_WT_F	2565	-	-	3'_DR+TG_Comm_R	3218	12718	-	653	no amplicon
3' KI	3'_DR+TG_KI_F	-	11638	-	3'_DR+TG_Comm_R	3218	12718	-	1080	100% match by Sanger sequencing
Flp Check	DR+TG_FlpChk_F	-	-	2463	DR+TG_FlpChk_R	-	-	2836	374	100% match by Sanger sequencing

Figure S6. Overview of genotyping results confirming the successful introduction of the DR+TG insert into the 3' region of murine *CTSD* via CRISPR-Cas. **A**, Genomic structure of the mouse *CTSD* gene prior to modification (top), after introduction of the DR+TG knockin (KI) allele (middle), and after removal of the TG portion using Flp-recombinase (bottom). The approximate positions of genotyping primers are indicated (blue arrows). **B**, Table showing the gRNA sequences, the targeted endogenous sequences (with PAM sequences in red), the amino-acid sequence encoded by the sequence targeted by gRNA_3, and the modifications to gRNA-targeted regions within the KI targeting construct. **C**, Table of DNA primers used for genotyping. **D**, Summary of genotyping results, showing the relative positions of individual primers, the predicted amplicon sizes for primer pairs, and the outcome of PCR amplification and subsequence Sanger sequencing. These results apply to a single clonal cell line chosen for use in downstream experiments. Note that no amplification was obtained for WT amplicons.

Supplemental Methods.

Cloning methods for individual DNA constructs.

S1.1 Cloning of DNA constructs

S1.1.1 Vectors for transient transfection experiments

The parent vector expressing destabilized GFP (GFPd2) under the control of the CAG promoter, pCAG-GFPd2 (Addgene plasmid #14760 [1]) was modified by inserting an open-reading frame (ORF) encoding a puromycin resistance cassette (Puro^r)-T2A-TagBFP fusion protein (amplified from Addgene Plasmid #155307 [2]) into an AvrII restriction site between the SV40 promoter and SV40 poly(A) signal within the pCAG-GFPd2 vector. After sequence verification, de novo synthesized DNA sequences encoding one or more DRs for 5 different Cas RNases were inserted into a NotI site within the 3' UTR. The ORFs for dCas12a without or with nuclear localization signals (NLSs) were generated by PCR from pSLQ10844 (Addgene Plasmid #183956 [3]). ORFs for the remaining Cas RNases (PfCas6, SsoCas6, CasE and Csy4) containing an N-terminal FLAG sequence (both without or with an adjacent NLS) were synthesized de novo, as mammalian codon-optimized gBlock DNA sequences based on amino acid sequences derived from Campa et al. [4]. The latter ORFs were inserted into the pICherryNeo vector (gift of Dario Vignali; Addgene Plasmid #52119) digested with XbaI.

2.1.2 Vectors for inducible expression of Cas RNases (and mCherry and Neo^r)

Vectors for inducible expression of Cas RNases (or no RNase) along with mCherry (mCh) with 3 C-terminal NLSs and neomycin/G418 resistance (Neo^r) were generated as follows. A de novo synthesized DNA sequence encoding (1) appropriate restriction sites for in-frame insertion of ORFs for Cas RNases, (2) a P2A sequence, (3) mCh, (4) a T2A sequence, (5) Neo^r, and (6) overlapping sequences was cloned into the EcoRI and NotI sites within pTet-One [5] (Takara Bio USA, Inc., San Jose, CA, USA) to create pMT_mCh_NeoR_pT1. The ORF for dCas12a (the codon-optimized version) containing 2 NLSs was amplified from pSLQ10875 (Addgene Plasmid #183962 [3]) and the ORFs for PfCas6, SsoCas6, CasE and Csy4 with NLSs were amplified from the pICherryNeo vectors described above and cloned into NcoI and SacI sites within pMT_mCh_NeoR_pT1.

S1.1.3. Targeting construct for inserting 3 Cas12a DRs into the 3' UTR of the murine CTSD

HiFi DNA Assembly was used to assemble the following in pBlueScript KS II(+) (Agilent Technologies, Santa Clara, CA, USA) digested with NotI and KpnI: (1) a ~750-bp 5' homology arm containing overlap with the vector on the 5' end, a LoxP site on the 3' end, and modifications to the sequences within the *CTSD* ORF recognized by gRNAs used for insertion (see Supp. Fig. S2), PCR amplified from C57Bl/6J mouse tail DNA; (2) a PCR amplicon of the IRES from pICherryNeo with appropriate 5' and 3' overhangs; (3) the ORF for Puro^r_P2A_GFP and a second LoxP site, amplified from Addgene Plasmid #111596 [6]; (4) a PCR amplicon of the 3 Cas12a DRs, amplified from that cloned into in pCAG-GFPd2; and (5) a ~750-bp 3' homology arm, with overlap for the pBlueScript vector, amplified from C57Bl/6J mouse tail DNA.

S1.1.4. Targeting construct for the DR+TG insert

The region encoding the artificial intron, the ORF for dCas12a, the IRES and the mCherry ORF was PCR amplified from the previously generated vector encoding dCas12a in pICherryNeo and cloned into pTet-One digested with EcoRI and NotI. The resulting vector, pdCas12a_pICNT, was digested with BamHI and NotI, and a PEST sequence, amplified from pCAG_GFPd2, was inserted in-frame into the C-terminus of dCas12a, to generate pdCas12a_PEST_pICNT. The MultiSite Gateway Pro kit was used to assemble the final construct, from 3 pieces combined into a fourth pDEST vector, according to manufacturer's recommendations (Thermo Fisher Scientific, Waltham, MA, USA). The first piece was initially constructed in pBlueScript KS II(+) cut with SacI and XhoI, into which was inserted two de novo synthesized gBlocks encoding: (1) a synthetic gRNA (syn_gRNA) [7] for ultimate excision of the construct for CRISPR; (2) a ~600 bp homology arm, with substitutions to remove the gRNA sequence used for modifying the *CTSD* allele; (3) a Cas12a DR flanked on the 5' end by the synSeparator sequence (AAAT; [8]), positioned immediately downstream of the stop codon (see Supp. Fig. S3); (4) the endogenous *CTSD* 3' UTR; and (5) an FRT sequence. Once assembled, the latter elements were amplified using primers containing appropriate attB sequences, then introduced into pDONR221 P1-P4 using BP Clonase II (Thermo Fisher Scientific, Waltham,

MA, USA). The second piece was constructed by PCR amplifying a region encoding (1) the tetracycline response element (TRE); (2) the artificial intron; (3) the dCas12a with C-terminal PEST sequence; (4) the IRES; (5) the mCherry ORF; and (6) the poly(A) sequence, from pdCas12a_PEST_pICNT, using primers containing appropriate attB sequences, which was then introduced (in reverse orientation) into pDONR221 P4r-P3r. The third piece was generated by PCR amplifying, with primers containing appropriate attB sequences, a de novo synthesized gBlock encoding a ~600-bp 3' homology region downstream of the *CTSD* 3' UTR followed by a second Syn_gRNA sequence. This sequence was introduced into pDONR221 P3-P2 using BP Clonase, then the CAG promoter, obtained from pCAG-GFPd2 as a SalI-SalI fragment was subsequently introduced (also in reverse orientation) into a SalI site within the latter gBlock. After sequence verification, the latter three vectors were assembled into pcDNA6.2™/V5-pL-DEST using LR Clonase II (Thermo Fisher Scientific, Waltham, MA, USA), generating a construct called pCTSD_DR+TG.

Supplemental References

1. Matsuda, T., and C. L. Cepko. "Controlled expression of transgenes introduced by in vivo electroporation." *Proc Natl Acad Sci U S A* 104, no. 3 (2007): 1027-32.
2. Abbott, T. R., G. Dhamdhere, Y. Liu, X. Lin, L. Goudy, L. Zeng, A. Chemparathy, S. Chmura, N. S. Heaton, R. Debs, T. Pande, D. Endy, M. F. La Russa, D. B. Lewis, and L. S. Qi. "Development of CRISPR as an Antiviral Strategy to Combat SARS-CoV-2 and Influenza." *Cell* 181, no. 4 (2020): 865-76 e12.
3. Guo, L. Y., J. Bian, A. E. Davis, P. Liu, H. R. Kempton, X. Zhang, A. Chemparathy, B. Gu, X. Lin, D. A. Rane, X. Xu, R. M. Jamiolkowski, Y. Hu, S. Wang, and L. S. Qi. "Multiplexed genome regulation in vivo with hyper-efficient Cas12a." *Nat Cell Biol* 24, no. 4 (2022): 590-600.
4. Campa, C. C., N. R. Weisbach, A. J. Santinha, D. Incarnato, and R. J. Platt. "Multiplexed genome engineering by Cas12a and CRISPR arrays encoded on single transcripts." *Nat Methods* 16, no. 9 (2019): 887-93.
5. Loew, R., N. Heinz, M. Hampf, H. Bujard, and M. Gossen. "Improved Tet-responsive promoters with minimized background expression." *BMC Biotechnol* 10 (2010): 81.
6. Horlbeck, M. A., A. Xu, M. Wang, N. K. Bennett, C. Y. Park, D. Bogdanoff, B. Adamson, E. D. Chow, M. Kampmann, T. R. Peterson, K. Nakamura, M. A. Fischbach, J. S. Weissman, and L. A. Gilbert. "Mapping the genetic landscape of human cells." *Cell* 174, no. 4 (2018): 953-67 e22.
7. Ishibashi, R., K. Abe, N. Ido, S. Kitano, H. Miyachi, and F. Toyoshima. "Genome editing with the donor plasmid equipped with synthetic crRNA-target sequence." *Sci Rep* 10, no. 1 (2020): 14120.
8. Magnusson, J. P., A. R. Rios, L. Wu, and L. S. Qi. "Enhanced Cas12a multi-gene regulation using a CRISPR array separator." *Elife* 10 (2021).
